# Layer-Dependent Mechanical Properties and Enhanced Plasticity in the Van der Waals Chromium Trihalide Magnets

**DOI:** 10.1021/acs.nanolett.0c04794

**Published:** 2021-04-09

**Authors:** Fernando Cantos-Prieto, Alexey Falin, Martin Alliati, Dong Qian, Rui Zhang, Tao Tao, Matthew R. Barnett, Elton J. G. Santos, Lu Hua Li, Efrén Navarro-Moratalla

**Affiliations:** ■ Instituto de Ciencia Molecular, Universitat de València, Calle Catedrático José Beltrán Martínez 2, 46980, Paterna, Spain; ‡ Guangdong Provincial Key Laboratory of Functional Soft Condensed Matter, School of Materials and Energy, Guangdong University of Technology, Guangzhou 510006, China; § Institute for Frontier Materials, Deakin University, Geelong Waurn Ponds Campus, Waurn Ponds, Victoria 3216, Australia; ∥ School of Mathematics and Physics, Queen’s University Belfast, BT7 1NN Belfast, United Kingdom; ⊥ Department of Mechanical Engineering, The University of Texas at Dallas, Richardson, Texas 75080, United States; # Institute for Condensed Matter Physics and Complex Systems, School of Physics and Astronomy, The University of Edinburgh, EH9 3FD Edinburgh, United Kingdom; ¶ Higgs Centre for Theoretical Physics, The University of Edinburgh, EH9 3FD Edinburgh, U.K.

**Keywords:** 2D magnetic materials, mechanical properties, strain tunability, nanoindentation, Young’s modulus, plasticity

## Abstract

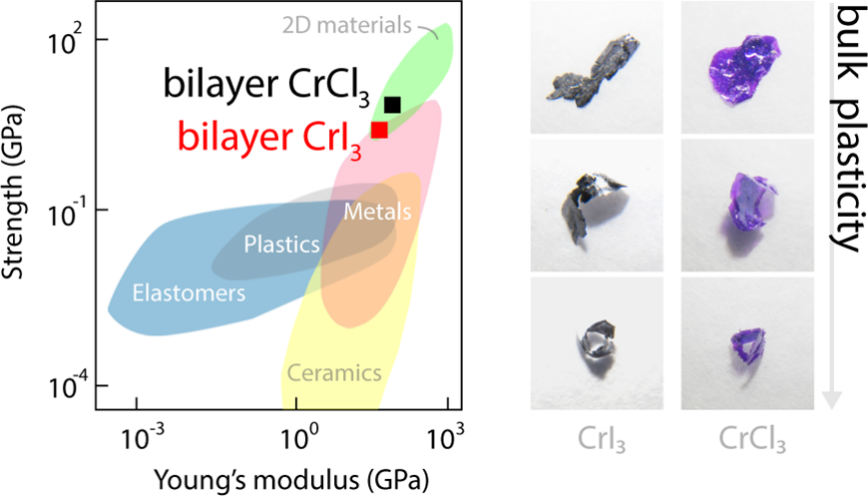

The mechanical properties of magnetic materials are instrumental for the development of magnetoelastic theories and the optimization of strain-modulated magnetic devices. In particular, two-dimensional (2D) magnets hold promise to enlarge these concepts into the realm of low-dimensional physics and ultrathin devices. However, no experimental study on the intrinsic mechanical properties of the archetypal 2D magnet family of the chromium trihalides has thus far been performed. Here, we report the room temperature layer-dependent mechanical properties of atomically thin CrCl_3_ and CrI_3_, finding that the bilayers have Young’s moduli of 62.1 and 43.4 GPa, highest sustained strains of 6.49% and 6.09% and breaking strengths of 3.6 and 2.2 GPa, respectively. This portrays the outstanding plasticity of these materials that is qualitatively demonstrated in the bulk crystals. The current study will contribute to the applications of the 2D magnets in magnetostrictive and flexible devices.

The magnetic moment of a crystal is susceptible to the application of external strain,^1^ as a consequence magnetostriction has had a big technological relevance in the past century.^2−4^ The recent isolation of free-standing 2D magnets,^5−9^ has settled long-standing fundamental questions^7^ and enabled ultrathin magnetoelectric devices.^10−12^ However, despite recent works that have demonstrated a strong modulation of 2D magnetism in atomically thin CrI_3_ under high-pressure values,^13,14^ direct-strain modulation has only been attempted for <0.3% strain values,^15^ and the prospects of the modulation of magnetism in the 2D limit have therefore not been fully explored. This can be attributed to a lack of fundamental understanding of the intrinsic mechanical properties of 2D magnets, which proves vital to realize their various applications. Indeed, although CrCl_3_ was first studied by Kamerlingh Onnes^16^ at the beginning of the last century, no experimental data on the mechanical properties of the magnetic chromium trihalide (CrX_3_, X = I, Cl, Br) bulk or few-layer crystals has been reported to date.

The mechanical properties of 2D materials have been shown to be different from those of their bulk counterparts. Graphene, for instance, has Young’s modulus of ∼1 TPa and breaking strength of 130 GPa,^17^ significantly higher than in graphite.^18,19^ A similar trend has been observed in other 2D materials, such as atomically thin hexagonal boron nitride (hBN) (0.87 TPa in Young’s modulus and 70 GPa in breaking strength) and molybdenum disulfide (MoS_2_) (0.33 TPa in Young’s modulus and 30 GPa in breaking strength).^20,21^ It is worth mentioning that these strength values are far beyond the yield strength measured in conventional materials (i.e., ∼3 GPa for that of silicon),^22^ demonstrating the capability of 2D materials to sustain an enormous strain without failure,^23^ for example, up to 25% in the case of graphene.^17^ On the other hand, the multilayer forms of these van der Waals crystals can benefit from their layered structure to achieve large plasticity. Such exceptional behavior has been recently reported in InSe, portraying this material as a strong candidate for near-future deformable electronics.^24,25^ It is therefore timely to explore the layer-dependent intrinsic mechanical properties of the chromium trihalides as archetypal magnetic 2D materials.

In our experiment, we obtained atomically thin CrI_3_ and CrCl_3_ flakes down to the bilayer (2L) by mechanical exfoliation of bulk crystals. The exfoliation was directly performed on substrates with prefabricated microwells for atomic force microscopy (AFM) nanoindentation (see Supporting Information for details). Figure 1a,d shows an optical micrograph of atomically thin CrI_3_ and CrCl_3_ covering several holes in a 90 nm-SiO_2_/Si substrate. Figure 1b and e shows the corresponding AFM images in contact mode, portraying a thickness of 1.7 and 1.4 nm (Figure 1c,f), respectively, which corresponds to 2L CrCl_3_ and CrI_3_.

**Figure 1:**
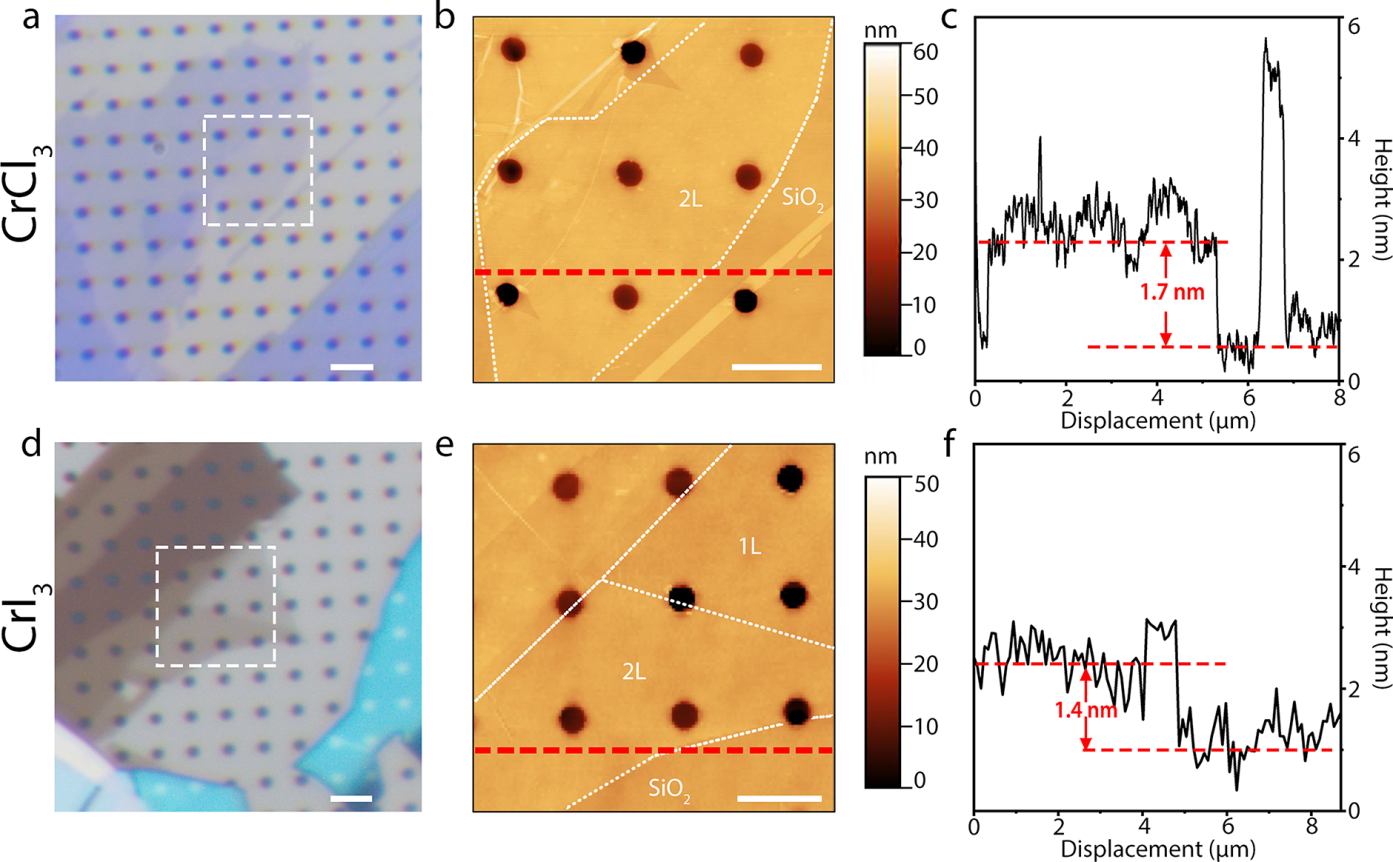
Characterization of CrX_3_ nanosheets. (a, d) Optical microscopy image of 2L and few-layers of CrCl_3_ and CrI_3_ crystals, respectively, on a SiO_2_/Si substrate suspended over microwells of 600 nm in diameter; (b, e) AFM image of the CrCl_3_ and CrI_3_ thin crystals, respectively, corresponded to the square area of optical images (a,d, respectively); and (c, f) the corresponding height traces of the dashed line in panels b and e of 2L CrCl_3_ and 2L CrI_3_ crystals, respectively. Scale bars in white, 3 μm in panels a and d and 2 μm in panels b and e.

The mechanical properties of the few-layer CrI_3_ and CrCl_3_ were probed by the nanoindentation technique performed with the same AFM used for topographic inspection.^17,20^ The load–displacement curves were obtained by applying a load at the center of each suspended region until fracture for a minimum of five indentations per thickness per material to ensure the repeatability of the results. The curves were then fitted by a well-established model^17^ (see section 4 of the Supporting Information) as demonstrated in Figure 2a. From these results, we extracted Young’s modulus (*E*) for both materials in terms of their layer count (Figure 2b). The breaking strength (σ) (Figure 2c) and ultimate strain values were determined based on the obtained fracture loads and the load–displacement relationships by means of finite element simulation (FEM) (see section 5 in the Supporting Information). The volumetric Young’s modulus and breaking strength of 2L CrI_3_ and 2L CrCl_3_ were *E* = 43.4 ± 4.4 GPa and σ = 2.2 ± 0.5 GPa and *E* = 62.1 ± 4.8 GPa and σ = 3.6 ± 0.4 GPa, respectively. The ultimate strain was found directly under the tip and its values for 2L CrI_3_ and 2L CrCl_3_ were 6.09% and 6.49%, respectively. A direct comparison between the two materials indicates that both the Young’s modulus and the breaking strength of CrCl_3_ were larger than those of CrI_3_, depicting that the chromium trihalide materials with a heavier halide exhibit a lower mechanical stiffness. This trend correlates nicely with the ionic character of the Cr–X bond, which is stronger in the Cr–Cl interaction compared to Cr–I.^26^

**Figure 2:**
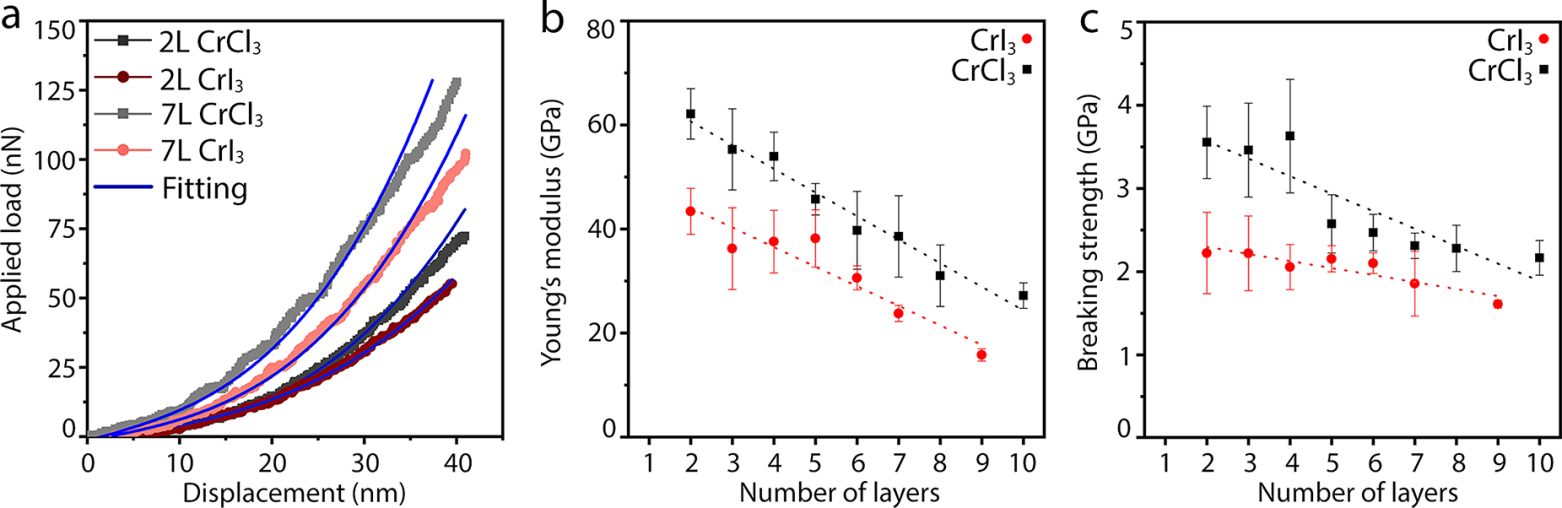
Mechanical properties of CrX_3_ nanosheets. (a) Load–displacement curves and the corresponding fittings for 2L,7L for CrCl_3_ and CrI_3_; (b) volumetric Young’s modulus; and (c) breaking strength of CrCl_3_ and CrI_3_ crystals of different thicknesses, along with dashed lines that show the linear fit of experimental Young’s moduli and breaking strength values.

Remarkably, as the thickness of the flakes increases, both atomically thin materials show a drop in Young’s modulus and breaking strength. For example, 9L CrI_3_ had *E* = 15.8 ± 1.2 GPa and σ = 1.6 ± 0.04 GPa, representing a 64% and 27% decrease in Young’s modulus and breaking strength compared to 2L, respectively. Similar trends were observed in CrCl_3_, where 10L CrCl_3_ had *E* = 27.1 ± 2.5 GPa and σ = 2.2 ± 0.2 GPa. To provide further insights into the layer-dependent mechanical properties of the chromium trihalides, we have undertaken van der Waals-corrected density functional theory (vdW-DFT) calculations to unveil the energy landscape of interlayer sliding shifts during the mechanical tests (see section 6 in the Supporting Information for details). Figure 3a shows a schematic of the atomic structure of bilayer CrX_3_ along the crystallographic *c* axis (direction of the AFM tip motion during indentation) in the monoclinic phase (space group *C*2/*m*) present at room temperature, with the definition of the two interlayer sliding paths utilized in the simulations: along [100] and along [010]. We apply a fractional lateral shift on one chromium-trihalide layer relative to the other starting from the AB stacking order (Figure 3b,c). The FEM simulations predict that the suspended 2L CrX_3_ crystals are mostly under small in-plane strain in the area far from the contact region even under the fracture loads. This picture changes in the region close to the indentation center where out-of-plane compression starts to play a key role in the fracture mechanism. Figure 3d and 3g shows the in-plane strain (solid lines) and out-of-plane compression (dashed lines) distributions close to the indentation center under different fracture loads for 2L CrCl_3_ and 2L CrI_3_, respectively. On the vdW-DFT calculations, three distinct regions were chosen to evaluate the sliding energy barriers. In the region far away from the indentation center, the equilibrium interlayer interaction occurs at 0 GPa out-of-plane compression and 0% in-plane strain (i.e., 0 GPa and 0% for both CrCl_3_ and CrI_3_). This choice of strain conditions is a valid approximation to our experiments, where extremely low values of strain, <0.5%, are found at the membrane edges (see Figure S3). The area just outside of the contact region is under a large in-plane strain but without any out-of-plane compression (5.35% and 0 GPa for CrCl_3_; 4.79% and 0 GPa for CrI_3_). The tip contact region experiences the highest in-plane strain and out-of-plane compression under the fracture loads (0.49 GPa and 6.49% for CrCl_3_; 0.36 GPa and 6.09% for CrI_3_). Figure 3e, 3f, 3h, and 3i summarizes the sliding energy per formula unit obtained for CrI_3_ and CrCl_3_ at different values of interlayer pressure and in-plane strain as provided by FEM simulations. In the regions of the membranes beyond ∼8.5 nm from the indentation centers (see Table S2), where no pressure and small strain are present in the systems, the individual layers of 2L CrI_3_ and CrCl_3_ tend to slide over each other despite the path considered, that is, [100] or [010] (Figure 3e–f and 3h–i). This process is mediated by thermal fluctuations (*kT* = 25.7 meV), which are present at room temperature. The interlayer barriers are below *kT* for the majority of the positions with the only exception at the fractional shift of 2/3. At this crystallographic position, there is a slight increment of the energy above *kT*, which prevents further sliding along both [100] and [010]. This indicates that the layers can displace almost freely with little energetic opposition (Figure 3f and 3i). As pressure and strain are applied (see Table S2), there is an increment of the energetic barriers at 2/3 along [100] for CrCl_3_ (168 meV) and CrI_3_ (209 meV) which indicates that the layers may find difficulties to slide over at that particular position (Figure 3e and 3h). The main driving force for such enhancement of the energies is the strong overlap of the charge density at 2/3 (Figure S5). Conversely, along [010] at 0.49 GPa and 6.49%, and 0.36 GPa and 6.09% for both CrCl_3_ and CrI_3_, respectively, the energies at 2/3 and their multiple positions (0, 1/3, 1) are below kT (Figure 3f and 3i) but slightly increments at intermediate positions (1/6, 1/2, 5/6) although still smaller than the barrier at 2/3 along [100] at finite strain and pressure. This suggests that the layers may choose a combination of sliding paths to minimize their energies as the pressure is applied. That is, the layers may start along the path [100] but may change direction to [010] to minimize their energies. Since the sliding path from 1/2 to 2/3 along [100] is symmetrically the same as along [010] (see Figure S7), the layers will follow a downhill energy profile from ∼76 (CrCl_3_) and ∼98 meV (CrI_3_) to 0 meV on both cases rather than increase their energy following the same path along [100]. These results are consistent with the variation of mechanical properties versus the number of layers which follows our previous analysis on graphene and hBN^20^ providing a plausible explanation for the layer-dependence of the mechanical properties in CrX_3_. It is worth mentioning that the energetic barriers observed in the sliding of the layers are particularly sensitive to the relaxation of the atoms involved (Cr, Cl, I) during the computation. Figure S8 shows results without any relaxation in the layers, which resulted in larger barriers. Indeed, lower energies than those shown in Figure 3e–f and Figure 3h–i can be achieved when the relaxation of the Cr atoms is also taken into account (see Figure S9) with a consequent expansion of the interlayer distance (see Figure S10). This correlates well with the positions where the energies increase during the sliding and suggests that changes of stacking order should be followed by expansion or contraction of the interlayer distance as recently measured.^14^ In addition, the magnitudes of the barriers for CrI_3_ are moderately larger than those for CrCl_3_, which suggests a slightly more stable dependence of the mechanical properties with the thickness, in agreement with the overall experimental trend observed.

**Figure 3:**
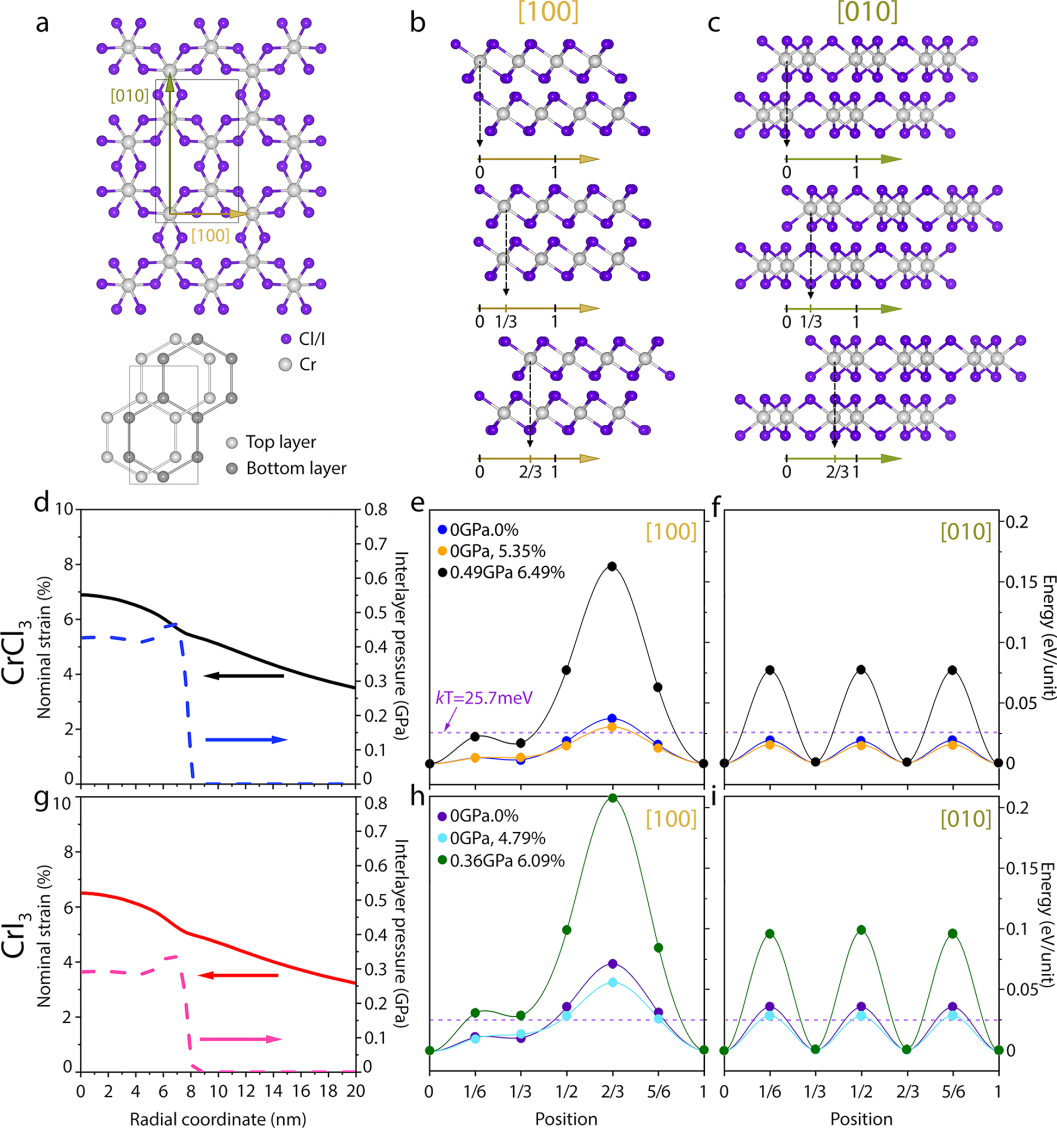
Sliding energies of bilayer CrX_3_ under different in-plane strain and out-of-plane compression conditions. (a) Top-view of the bilayer structure utilized in the vdW-DFT simulations, respectively. Two high-symmetry directions along the [100] and [010] are considered as representatives of the lateral sliding process occurring in the structures. Only Cr atoms are shown in a bottom to highlight the bilayer stacking. (b, c) Different positions correspond to fractional lateral shifts of the top-layer relative to the original AB stacking in units of [1/6, 0] and [1/6, −1/6] over the unit cell along [100] and [010], respectively. (d) FEM calculations of nominal strain (solid line) and interlayer pressure (dashed line) in 2L CrCl_3_ within a radial distance of 20 nm from the indentation center, where three distinct regions were chosen to study sliding energy per formula unit (eV/unit) of bilayer CrCl_3_ along (e) [100] and (f) [010], respectively. The sliding energy was determined with the Cr atoms being fixed and the Cl atoms being relaxed in the simulation (see Figures S8–S10 for additional details). The dashed line indicates available thermal-energy at room temperature (*kT* = 25.7 meV). Colored dots are the calculated vdW-DFT energies with a cubic interpolation (solid lines) between different positions. (g–i) Analogous analysis as in panels d–f for 2L CrI_3_. The monoclinic (space group *C*2/*m*) stacking order was utilized in all simulations.

Overall, the measured mechanical values are among the smallest ones observed within the family of 2D materials, that is, much less stiff than 2D transition metal dichalcogenides and mica.^21,27,28^Figure 4 shows a map of the mechanical properties of atomically thin CrX_3_ compared to other materials. The position of CrX_3_ on this chart can be qualitatively explained by taking into account the bonding energies inside of the crystal, which scale according to the magnitudes of Young’s modulus and breaking strength. While the dissociation energy for the honeycomb of C atoms in graphene yields a value of 805 kJ/mol,^29^ our DFT calculations indicate a formation energy of 260.9 kJ/mol for CrI_3_ and 597.7 kJ/mol for CrCl_3_, depicting a weaker interatomic interaction than that of the graphene lattice. In addition, within the chromium halide family, the smaller the ionic character the larger the bond energies,^26^ with a variation of the electronic localization function across the different Cr-halides.^30^ These results underline the soft nature of the chromium trihalides, which makes them extremely sensitive to small stress changes and very effective for strain modulation.

**Figure 4:**
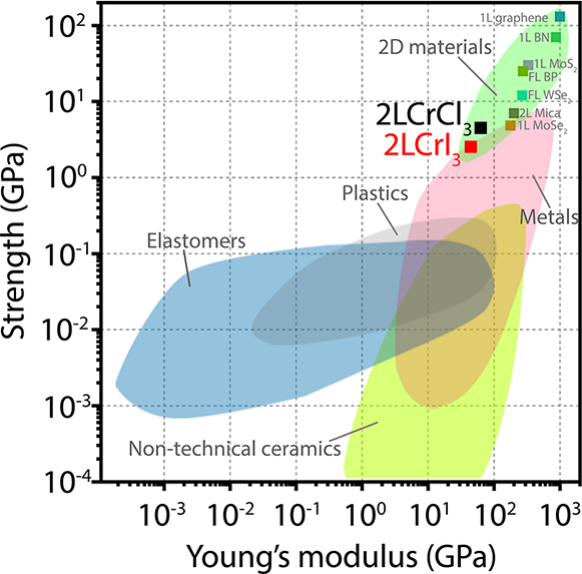
Map of mechanical properties of different materials in Young’s modulus—Ultimate/breaking strength space. The mechanical properties of different types of materials, including 2D crystals and measured here 2L of CrX_3_, are compared.

Although these results place the chromium trihalides as one of the softest 2D materials that have been experimentally measured so far, their breaking strength up to 10L is larger than that of silicon (∼2 GPa),^22^ showcasing the general outstanding mechanical properties of 2D materials. It is also important to consider the significance of the presence of imperfections in the crystals, which affects the elastic behavior. Griffith described how the breaking strength in brittle materials (see also section 7 on the Supporting Information) is governed by defects and imperfections,^31^ establishing a limit of σ ∼ *E*/9 by experimental extrapolation. In the limit of an ideal material, mechanics are governed by its molecular tensile strengths. In both chromium trihalides, the bilayers (σ ∼ *E*/20) and the multilayers (σ ∼ *E*/10) follow a behavior close to this limit. These results suggest that the mechanical behavior in CrX_3_ thin crystals is determined by the interatomic interactions rather than defects, indicating a high crystallinity and a low density of impurities in the suspended regions. In comparison, polycrystalline classical materials like silicon^22^ or tungsten alloys,^32^ σ < *E*/100, report much lower values.^31^ The nonlinear elastic constitutive behavior was assumed for modeling CrX_3_ few layer crystals in FEM, and the derived maximum strains are close to ∼6–6.5% for the bilayers (see section 5 on the Supporting Information). The prospects of the combination of the exceptional flexibility and strengths with the intrinsic magnetism of atomically thin CrX_3_ nature hold promise for an enhanced strain-tunability in ultrathin magneto-mechanical devices.^33^

Considering the remarkable flexibility of few-layer CrCl_3_ and CrI_3_, and the interlayer sliding origin of the layer-dependent Young’s moduli, we investigated the plastic behavior of the two magnetic van der Waals materials in their bulk form. This property is of great relevance for future flexible devices, and it has recently been observed in bulk crystals of InSe.^25^ The deformability factor (Ξ) proposed by Wei T-R et al. can be useful as a way to frame the plastic behavior of a material, it is related to the sliding (*E*_s_) and cleavage (*E*_c_) energies of layered materials via
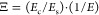
1where E is the volumetric Young’s modulus. The magnitudes of Ξ for bulk CrCl_3_ and CrI_3_ are plotted in Figure 5a and 5b as a function of the Young modulus and bandgaps for different materials with different electronic properties (semiconducting, insulators and metals). The cleavage energies were defined as the energies to separate bilayer CrX_3_ systems to two monolayers (see Figure S6), the sliding energies were taken from the most energetically favored sliding path in the equilibrium state, that is, along [010] direction (Figure 3), and the Young’s modulus values were extracted from the experimental data for 2L CrCl_3_ and CrI_3_. For both bulk CrCl_3_ and CrI_3_ the cleavage energies are larger than their sliding energies (see Table S3). Interestingly, the magnetic CrX_3_ showed one of the highest deformability factors of the 2D materials, even larger than that of the recently reported InSe (Figure 5).^25^ This outstanding capability for deformation is experimentally illustrated by macroscopically folding bulk CrI_3_ and CrCl_3_ crystals in Figure 5c. Upon further testing, CrX_3_ multilayered crystals could be confirmed to exhibit a superplastic behavior, which would open the door for their use in easily deformable and flexible devices that incorporate intrinsic magnetism.

**Figure 5:**
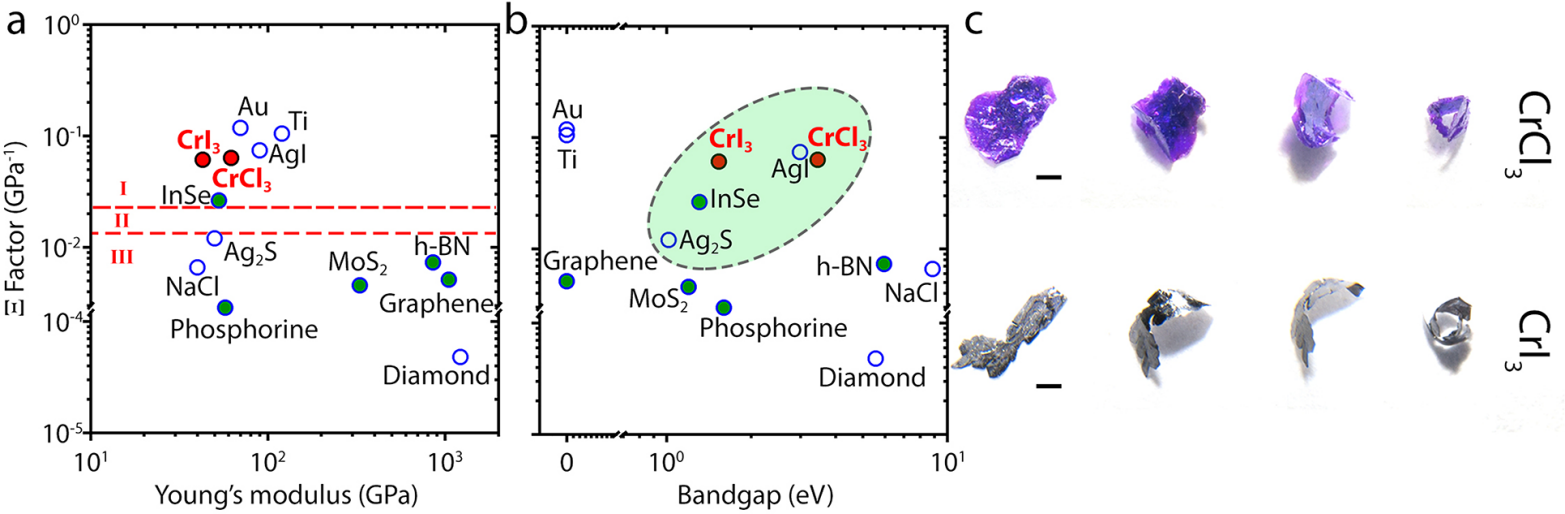
Deformability factor and enhanced plastic behavior of multilayer CrX_3_. (a) Deformability factor dependence on the Young Modulus, Regions I, II, and III correspond to plastic-flexible, potentially deformable, and brittle-rigid regions, respectively. The layered van der Waals materials are shown as green symbols; our experimental results are shown in red. (b) Deformability factor dependence on the bandgap for the same materials as panel a. The dashed line encircled green area are materials that show exceptional plastic behavior. The bandgaps for CrCl_3_ and CrI_3_ nanosheets are 3.44 and 1.53 eV, respectively.^33^ Panel c shows a folding sequence of flat bulk crystals of CrCl_3_ (top strip) and CrI_3_ (bottom strip) into a ring-like structure (enlarged in Figure S12). The scale bars are 1 mm.

## Supporting Information Available

Details on the bulk crystal growth, the fabrication of suspended atomically thin crystals, the characterization of the AFM indenters, the finite element analysis, the ab initio calculations performed, and supporting results regarding the plasticity in bulk crystals of CrX_3_ (PDF)
