# Supplementary material for: Layer-Dependent Mechanical Properties and Enhanced Plasticity in the Van der Waals Chromium Trihalide Magnets
Source: Nano Lett. 2021 Apr 9;21(8):3379–85. doi: 10.1021/acs.nanolett.0c04794 (PMC8454994; doi:10.1021/acs.nanolett.0c04794)
Supplement: Supplementary file 1 — nl0c04794_si_001.pdf [file nl0c04794_si_001.pdf]

Supporting information

Layer-dependent mechanical properties and enhanced plasticity in the van der Waals chromium trihalide magnets

Fernando Cantos-Prieto[†1], Alexey Falin[†2,3], Martin Alliati[4], Dong Qian[5], Rui Zhang[5], Tao Tao[2], Matthew R. Barnett[3], Elton J. G. Santos[6,7], Lu Hua Li*[3], Efrén Navarro-Moratalla*[1]*

1. Instituto de Ciencia Molecular, Universitat de València, Calle Catedrático José Beltrán Martínez 2, 46980, Paterna, Spain.

2. Guangdong Provincial Key Laboratory of Functional Soft Condensed Matter, School of Materials and Energy, Guangdong University of Technology, Guangzhou 510006, China.

3. Institute for Frontier Materials, Deakin University, Geelong Waurn Ponds Campus, Waurn Ponds, Victoria 3216, Australia.

4. School of Mathematics and Physics, Queen's University Belfast, BT7 1NN, United Kingdom

5. Department of Mechanical Engineering, The University of Texas at Dallas, Richardson, Texas 75080, USA.

6. Institute for Condensed Matter Physics and Complex Systems, School of Physics and Astronomy, The University of Edinburgh, EH9 3FD, United Kingdom.

7. Higgs Centre for Theoretical Physics, The University of Edinburgh, EH9 3FD, UK

Contents

1. Crystal growth 4
2. Fabrication of suspended atomically thin crystals 6
3. Atomic force microscopy 7
4. Drum resonator model 8
5. Finite elements analysis 9
6. *Ab initio* simulations 11
7. Brittle nature of few-layer CrX_3 21
8. Enhanced plasticity in multilayer CrX_3 22

1. Crystal growth

The single crystals of CrI_3 were grown by chemical vapor transport. Chromium powder (99.996, Alfa-Aesar) and beads of iodine (anhydrous 99.999 %, Sigma-Aldrich) were mixed in a 1:3 ratio inside an argon atmosphere in a glovebox. 972 mg of the mixture were loaded into a silica ampoule with a length, inner diameter and outer diameter of 500 mm, 15 mm and 16 mm respectively. The ampoule was extracted from the glovebox with a ball valve covering the open end to prevent air exposure and then it was immediately evacuated using a turbomolecular pump down to $6 \cdot 10^{-6}$. Once the pressure stabilized, the close end was dipped in liquid nitrogen to prevent the sublimation of the iodide beads. The ampoule was then flame sealed and introduced into a three-zone furnace with the material in the leftmost zone. The other two zones were heated up from room temperature to 650°C in 1440 minutes and kept for 1620 minutes to minimize nucleation sites in the growth zone. Then, the leftmost side was heated up to 700°C in 180 minutes. The three-zone furnace had a temperature gradient of 700°C/650°C /675°C. Subsequently, the temperature was kept constant for 7 days and cooled down naturally. Phase purity was inspected by powder X-ray diffraction (Fig. S1). After the crystal growth, the quartz tube was transferred into an argon glove box to prevent hydration, where the crystals were first purified by sublimation to remove the halide excess. Crystals were ground into powder using a razor blade and inserted into a capillary to perform X-Ray diffraction analysis. The phase of the bulk crystals was confirmed by fitting the X-ray diffraction pattern to the patterns computed from the single crystal structures previously reported[1] (Figure S1). The fit of the X-ray pattern revealed a monoclinic crystal system with a C12/m1 space group consistent with the crystal structure previously reported for CrI_3 at room temperature (ICSD 251654). Crystals of $CrCl_3$ were commercially obtained from © Strem chemicals as Chromium(III) chloride, anhydrous (99.9%-Cr), CAS number: 10025-73-7. A representative CrI_3 grown crystal is shown in Figure S1 together with its XRD characterization and a commercial $CrCl_3$ crystal.

---

[1] Michael A. McGuire, Hemant Dixit, Valentino R. Cooper, and Brian C. Sales. Coupling of Crystal Structure and Magnetism in the Layered, Ferromagnetic Insulator CrI_3. Chemistry of Materials **2015** 27 (2), 612-620.

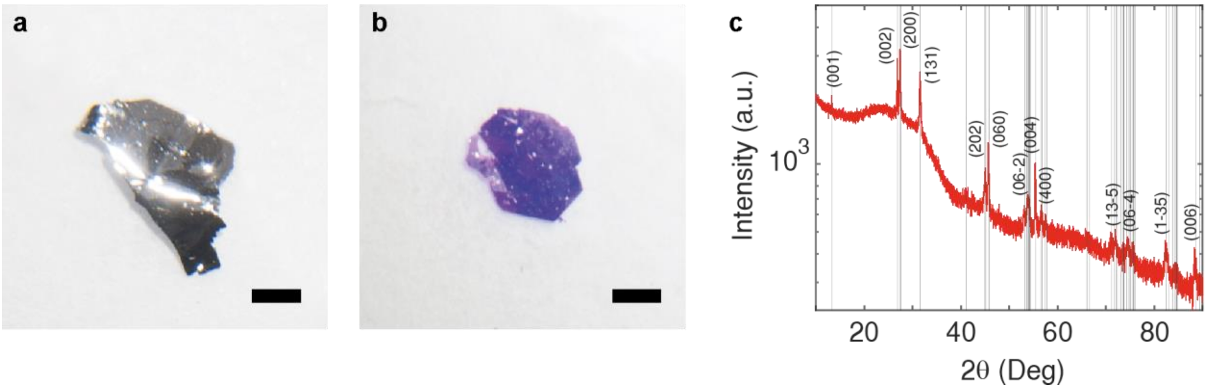

Figure S1. CrI_3 (a) and $CrCl_3$ (b) starting bulk crystals. Panel **c** shows the XRD powder pattern of CrI_3. The continuous red line corresponds to the measured diffraction points. Vertical lines indicate the theoretical positions of the Bragg diffraction peaks computed from the ICSD 251654 database[1], the miller indices of the main peaks are depicted next to them.

2. Fabrication of suspended atomically thin crystals

The crystals were cleaved using scotch tape to ensure a clean and flat starting surface. From this point, the materials were thinned down by successive mechanical exfoliation steps using a PDMS-based gel (Gel-Pak® PF film). The gel films were firstly inspected under the optical microscope for locating atomically thin flakes based on the optical contrast. Next, the flakes were transferred onto SiO_2/Si substrates with pre-fabricated micro-wells of 600nm in diameter, following a pressure-free procedure. That is, the gel film with the atomically thin flakes was placed on the substrate and, after fully engaged, it was slowly lifted up using a motorized arm. The procedure yielded atomically thin CrI_3 and $CrCl_3$ samples with a range of thicknesses down to the 2L. None of the 1L crystals found was suitable for the indentation experiments mainly due to their small surface area. The exfoliated samples were then transferred from the optical system to the AFM for topographic inspection. Note that both the sample preparation and AFM inspection were performed in the same chamber with an argon atmosphere to prevent the atmospheric degradation of the air-sensitive samples.

3. Atomic force microscopy

Topography imaging in tapping mode and nanoindentation in contact mode were conducted on the atomically thin CrX_3 using a Flex AFM from Nanosurf. These images were used to determine the thickness of the materials and center of the suspended regions of the CrX_3 for nanoindentation. Several silicon cantilevers (Tap300DLC) with a diamond-like-carbon coating on the tips were used to avoid deformation of the tip apexes under relatively high load. The spring constants of the cantilevers were determined using the thermal noise method. The tip radii were measured by scanning electron microscopy (Gemini SEM, see Figure S2). The indentations were performed on relatively large and clean flakes for more accurate results. The load-displacement curves with obvious hysteresis were excluded from further analysis. For accuracy purposes, particular care was taken in the selection of suitable suspended crystals free of contaminations and surface cracks by both optical observation and AFM scanning. The thermal drift of the samples was taken into consideration prior to commencement of the indentation process.

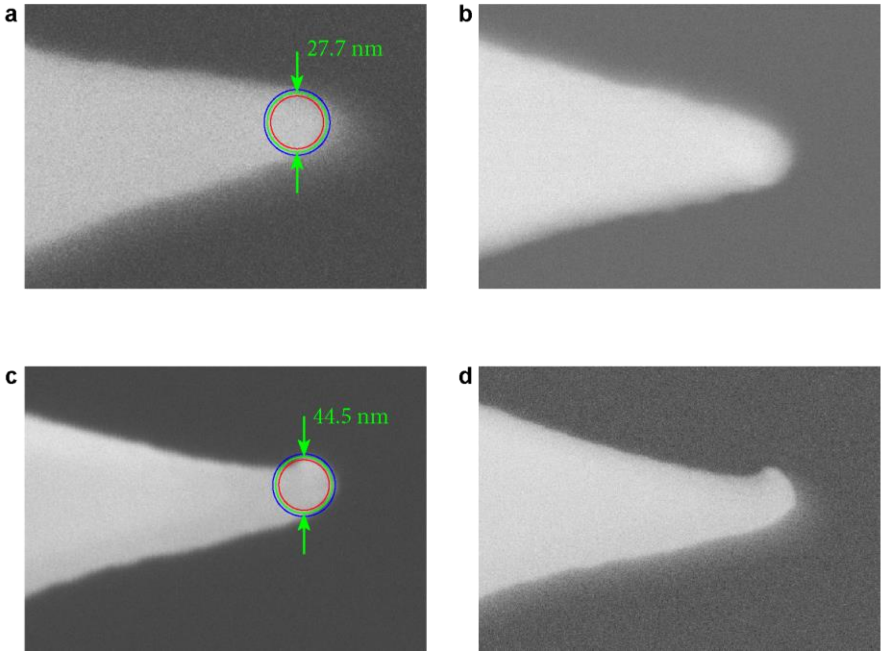

Figure S2. Two tips are shown before and after indentation. The first tip in panels **a,** before and **b,** after, and the second tip in **c,** before and **d,** after. The AFM indenter tip diameters were measured using a HRFESEM Zeiss GeminiSEM500. At the same time, the tips were measured before and after indenting, in order to ensure that the tip shape was not mechanically damaged or deformed.

4. Drum resonator model

The data extracted from the AFM indentations was fitted to a classical model for drum resonators which describes the load-displacement relation as:

$$F = {\sigma_0}^{2D}(\pi a)\left(\frac{\delta}{a}\right) + E^{2D}(q^3 a)\left(\frac{\delta}{a}\right)^3 \qquad (2)$$

where E^{2D} and ${\sigma_0}^{2D}$ are the effective Young's modulus and pre-tension of the CrX_3 flakes with the radius (a) of the suspended part, stretched with vertical displacement (δ) under the applied load (F); $q=1/(1.049-0.15\nu-0.16\nu^2)$ is a dimensionless coefficient related to the Poisson's ratio (ν), which are 0.253 and 0.297 for CrI_3 and $CrCl_3$, respectively[2]. This model describes the indentation behavior in classical drum resonators with low bending rigidity and small pre-tension. That is, a linear trend is considered for the membrane under small loads, while a cubic trend related to the stiffness of the membrane dominates when the load increases. The conventional bulk (*i.e.*, volumetric) Young's modulus *E* was calculated by dividing the 2D value, *i.e.* E^{2D} by the sample effective thickness, the used effective thicknesses of the CrI_3 and $CrCl_3$ were 0.662 nm and 0.611 nm, respectively[1, 3]. By using this model, we successfully extracted Young's modulus of CrX_3 for a range of thicknesses on both materials.

---

[2] Zhang, W.-B.; Qu, Q.; Zhu, P.; Lam, C.-H. Robust Intrinsic Ferromagnetism and Half Semiconductivity in Stable Two-Dimensional Single-Layer Chromium Trihalides. J. Mater. Chem. **2015**, 3 (48), 12457–12468.

[3] McGuire, M. A.; Clark, G.; Santosh, K. C.; Chance, W. M.; Jellison, G. E., Jr; Cooper, V. R.; Xu, X.; Sales, B. C. Magnetic Behavior and Spin-Lattice Coupling in Cleavable van Der Waals Layered $CrCl_3$ Crystals. Physical Review Materials **2017**, 1 (1), 014001.

5. Finite elements analysis

The analysis was performed using the commercial nonlinear finite element code ABAQUS. The $CrCl_3$ and $CrCl_3$ nanosheets were modeled as axisymmetric shells with a radius of 300 nm and the initial thicknesses are 0.6114*N nm and 0.6623*N nm, respectively, where N is the number of layers. The nanoindenters were modeled as rigid spheres with radii corresponding to those used in the experiment. The model employed two-node linear axisymmetric shell elements (SAX1), with mesh densities linear variation from 0.1 nm (center) to 5.0 nm (outermost) along the 300nm shell radius. The tip-top layer interaction was modeled by a frictionless contact algorithm. The displacement-controlled loading with a prescribed 0.1 nm per load step was applied to the spherical indenter. The nonlinear elastic constitutive behavior of both $CrCl_3$ and CrI_3 were assumed and it can be expressed under a uniaxial load as $\sigma = E\varepsilon + D\varepsilon^2$, where σ and ε are he the symmetric second Piola-Kirchhoff stress and the uniaxial Lagrangian strain, respectively. The Young's moduli (E) and the third-order elastic constant (D) values of $CrCl_3$ and CrI_3 were set to the values obtained from experimental results by using an analytical method. The nonlinear elastic behavior was implemented in ABAQUS using a previously described equivalent elastic-plastic material model[4]. The simulation loading steps corresponding to the point of membrane fracture were identified based on the fracture loads from the experiment. Therefore, fracture strength was derived as a volume average of the stress values of the elements that were directly underneath the nanoindenter at the loading step corresponding to the fracture point in the load-displacement curves obtained from the finite element methods. Full strain distributions along the membrane radius for both 2L $CrCl_3$ and CrI_3 are shown in Figure S3.

[4] Brown, W. F. Theory of Magnetoelastic Effects in Ferromagnetism. J. Appl. Phys. **1965**, 36 (3), 994–1000.

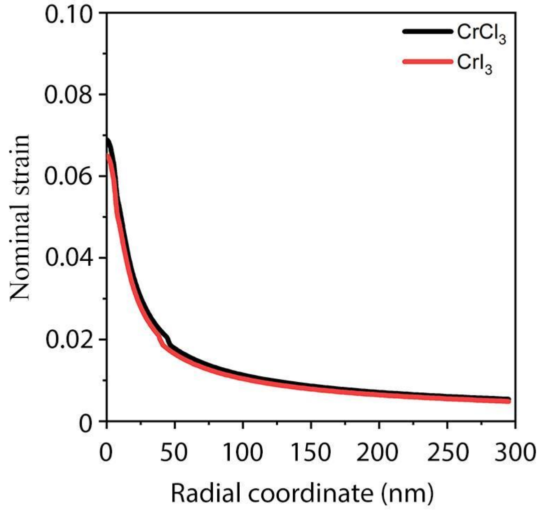

Figure S3. Strain distribution along the radius of the 2L $CrCl_3$ and CrI_3 nanosheets under the fracture load. These results were obtained from finite element simulations.

6. Ab initio simulations

Calculations were performed using Density Functional Theory as implemented in the VASP package[5]. Within the generalized gradient approximation (GGA), the Perdew-Burke-Ernzerhof (PBE)[6] exchange-correlation functional was used. For bilayer and bulk, Van der Waals interactions were taken into account through the many-body dispersion energy method[7]. A Hubbard correction was included through the Dudarev formulation[8]. The parameter *J* was fixed to 0.9 eV while the *U* energy was adjusted to obtain a U effective of 2.63 and 2.65 eV for $CrCl_3$ and CrI_3, respectively. The later U values were taken from the literature[9]. The electronic convergence criterion was set to $1x10^{-7}$ eV, while the structural optimizations were performed until all forces were lower than 0.001 eV/Å. All calculations were spin polarised. Energy cut-off of 800 eV, which is considerably higher than the maximum ENMAX in the pseudopotentials (288 eV) was utilized to ensure tight convergence. For orthorhombic (LT stacking) systems with no periodicity along the z direction (monolayer, bilayer and trilayer), the k-space was sampled *via* a 6x6x1 automatically generated Gamma-centred grid. Convergence with respect to k-point sampling was addressed at this point by comparison with the mechanical properties obtained with a 9x9x1 k-point grid. As the differences in the Young's moduli were negligible (~0.1%), the 6x6x1 sampling was used throughout the entire simulation set. The k-point grid was adjusted to keep the sampling density constant whenever the dimensions of the unit cell changed, i.e., 6x4x1 for monoclinic (HT stacking) bilayers and trilayers.

---

[5] Kresse, G. & Furthmüller, J. Efficient iterative schemes for ab initio total-energy calculations using a plane-wave basis set. Phys. Rev. B Condens. Matter. **1996**, 54, 11169–11186.

[6] Perdew, J. P., Burke, K. & Ernzerhof, M. Generalized Gradient Approximation Made Simple. Phys. Rev. Lett. **1996**, 77, 3865–3868.

[7] Tkatchenko, A.; Scheffler, M. Accurate Molecular van Der Waals Interactions from Ground-State Electron Density and Free-Atom Reference Data. Phys. Rev. Lett. **2009**, 102 (7), 073005.

[8] Dudarev, S. L.; Botton, G. A.; Savrasov, S. Y.; Humphreys, C. J.; Sutton, A. P. Electron-Energy-Loss Spectra and the Structural Stability of Nickel Oxide: An LSDA+U Study. Phys. Rev. B Condens. Matter **1998**, 57 (3), 1505–1509.

[9] Liu, J.; Sun, Q.; Kawazoe, Y.; Jena, P. Exfoliating Biocompatible Ferromagnetic Cr-Trihalide Monolayers. Phys. Chem. Chem. Phys. **2016**, 18 (13), 8777–8784.

We used two stackings in the simulations, namely rhombohedral (space group $R\underline{3}$) as observed at low temperature (LT) and monoclinic (space group C2/m) at high temperature (HT)[1]. Negligible variations were observed when the rhombohedral (space group $R\underline{3}$) was utilized[1, 3] (see Table S1). In terms of magnetic ordering, all monolayers were considered to be FM. The bilayer and trilayer were assumed intra-layer FM and inter-layer AFM, for both materials.

In order to calculate the mechanical properties, the following settings were used:

- IBRION = 6; allows for the calculation of the Hessian matrix
- ISIF = 3; stress tensor calculated – all degrees of freedom allowed to change
- NFREE = 4; number of displacements per direction per ion (more than this would be excessive, 2 would be ok but not optimum, 1 should be avoided). For trilayers, NFREE = 2 was required due to the computational cost.

These settings produce the 'TOTAL ELASTIC MODULI' in the OUTCAR file, namely, the matrix of elastic-stiffness coefficients 'C'. This is a 6x6 matrix relating the six independent stress and strain components (1 = XX, 2 = YY, 3 = ZZ, 4 = YZ, 5 = ZX and 6 = XY). With these coefficients, one can calculate the angle dependent in plane volumetric Young's Modulus, according to the equation[9, 10]:

$$E(\theta) = \frac{C_{11}\,C_{22} - {C_{12}}^2}{C_{11}\, sinsin\,\theta^{\,4} + C_{22} coscos\,\theta^{\,4} + \left(\frac{C_{11}\,C_{22} - {C_{12}}^2}{C_{44}} - 2\,C_{12}\right) sinsin\,\theta^{\,2} coscos\,\theta^{\,2}} \qquad (3)$$

Each of these coefficients is given volumetrically by VASP, i.e., in units of kBar. In the bulk case, they can be used directly. In the low dimensional cases, they have to be rescaled in order to 'remove' the vertical vacuum space in the unit cell. As the volume is in the denominator, the rescaling would imply multiplying the coefficient by the module of the third lattice vector (LV_3) projected over the vertical coordinate (Z) and dividing by the interlayer distance times the number of layers. The interlayer distance (d) is taken from our bulk optimized structure and used in the monolayer, bilayer and trilayer systems.

---

[10] Cadelano, E.; Palla, P. L.; Giordano, S.; Colombo, L. Elastic Properties of Hydrogenated Graphene. Phys. Rev. B Condens. Matter **2010**, 82 (23), 235414.

$$C_{rescaled} = C_{from\,VASP} \times \frac{\overrightarrow{LV_3} \cdot \check{z}}{d \cdot N_{layers}} \qquad (4)$$

As per our GGA+U calculations, the bulk interlayer distances are 5.859 and 6.698 Å for $CrCl_3$ and CrI_3, respectively.

	Young modulus (GPa)			
	Monoclinic (space group C2/m)		Rhombohedral (space group R$\underline{3}$)	
Layer number	$CrCl_3$	CrI_3	$CrCl_3$	CrI_3
1L	60.68	40.49	60.68	40.49
2L	59.51	38.35	60.01	36.57
3L	60.03	37.14	59.88	38.9

Table S1. **Young's Modulus at $\boldsymbol{\theta = 0°}$ (see Figure S3) versus the number of layers.** Different stacking sequences were utilized in the simulations such as rhombohedral (space group R$\underline{3}$) which was observed at low temperature (LT) and monoclinic (space group C2/m) a high temperature (HT)[1, 3].

CrI_3	Distance from the center, [nm]	0	8.5	300
	Max contact pressure, [GPa]	0.36	0.00	0.00
	In-plane strain, [%]	6.09	4.79	0.00
$CrCl_3$	Distance from the center, [nm]	0	8.5	300
	Max contact pressure, [GPa]	0.49	0.00	0.00
	In-plane strain, [%]	6.49	5.35	0.00

Table S2. **Values of strain and out-of-plane compression obtained by FEM.** These values of nominal strain and out-of-plane compression were obtained from FEM based on the experimental data, which has been used for sliding energy calculation by vdW-corrected density functional theory method.

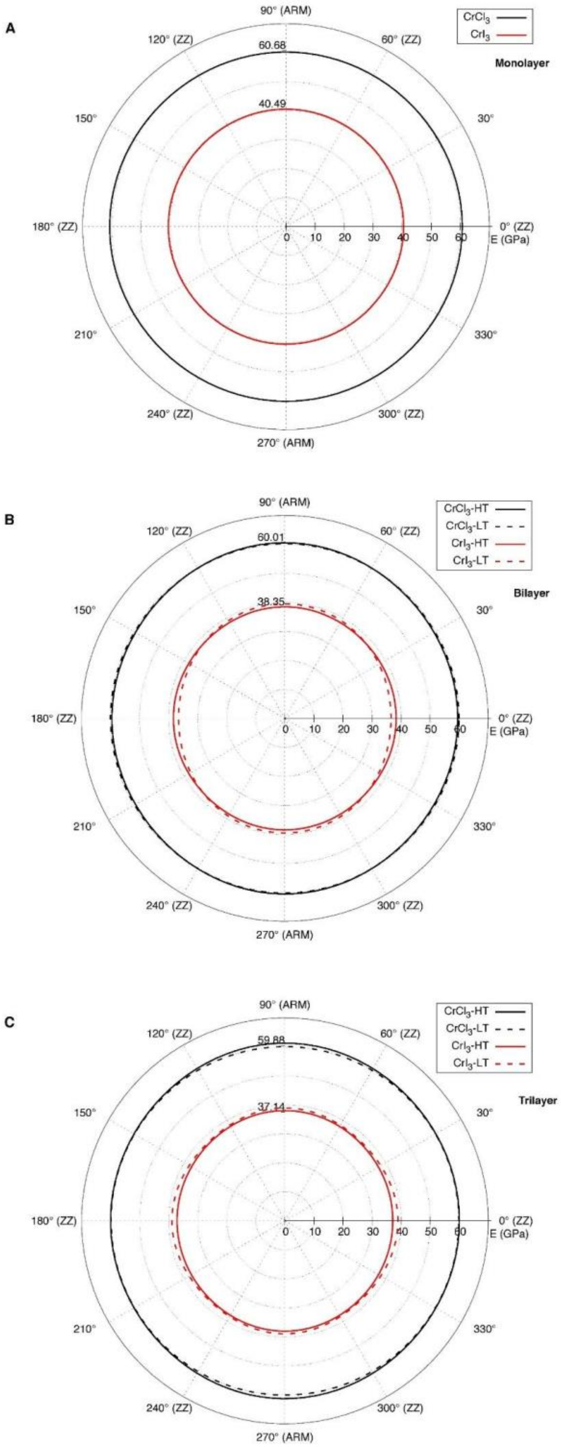

Figure S4. Polar plots θ (º) of the Young modulus E (in GPa) of monolayer (a), bilayer (n) and trilayer (c) CrX_3 (X=Cl, I) using two stacking sequences: rhombohedral (space group R$\underline{3}$) at low temperature (LT) and monoclinic (space group C2/m) a high temperature (HT)[1, 3]. Different orientations of the crystals along zigzag (ZZ) and armchair (ARM) are shown. Small anisotropies are observed throughout different directions.

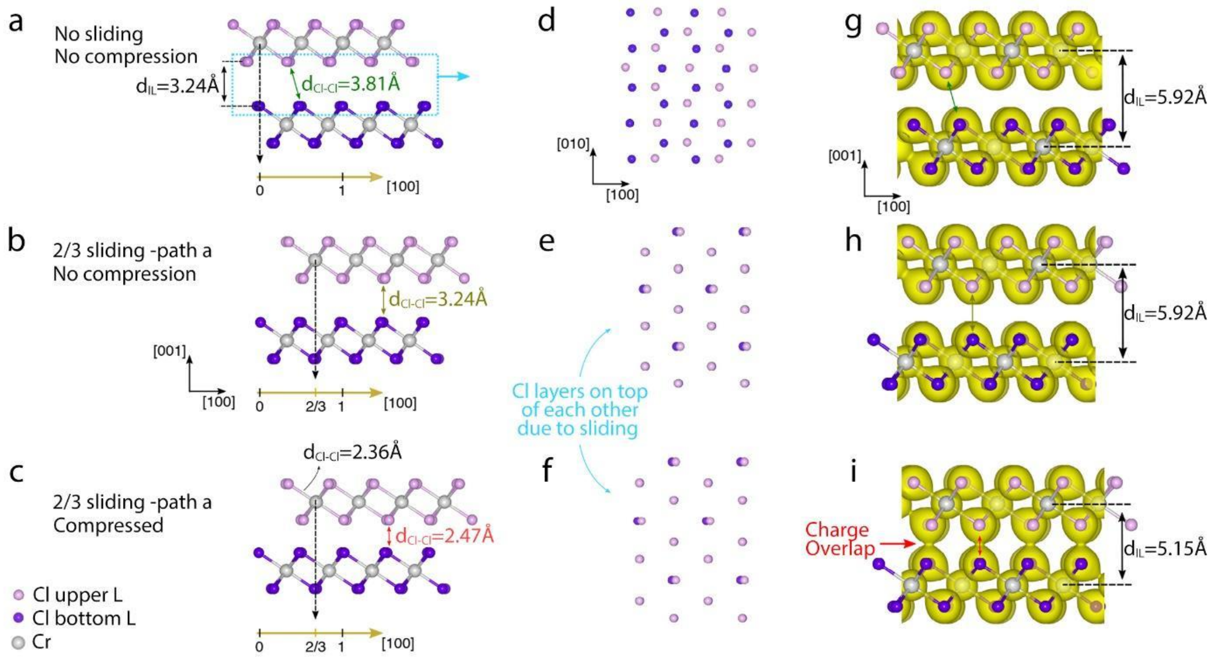

Figure S5. Charge density calculations of CrX_3. a-c, Side-, and **d-f,** top-views, respectively, of bilayer $CrCl_3$ at: **a, d,** the equilibrium position with no strain, pressure and displacement relative to the original stacking order; **b, e,** at 2/3 of the lateral shift relative to the origin at 0 along [100] with no compression; and **c, f,** at 2/3 position with applied compression. Cl atoms at the top and bottom layers are shown with distinct colors to highlight the shift. **g-i,** Charge density plots at the three situations with no compression or slide (**a, d**), with no compression but glided relative to the origin (**b, e**), and with compression (**c, f**), respectively. At 2/3 sliding along path-a ([100]) the chalcogenide atoms across the van der Waals (vdW) gap end up right on top of each other. That is, the lower Cl plane of the upper layer and the upper Cl plane of the lower layer have the same x and y coordinates. This results in the shortening of the Cl-Cl distance across the vdW gap and justifies why the energy increases in that particular sliding condition (in general, regardless of the pressure). This does not happen with other stackings or glidings. When hydrostatic pressure is applied in the system, the reduction of the interlayer distance brings the Cl atoms even closer. This ended up in covalent-like distances, and a strong overlapping charge density. Similar analysis applies for CrI_3 not shown.

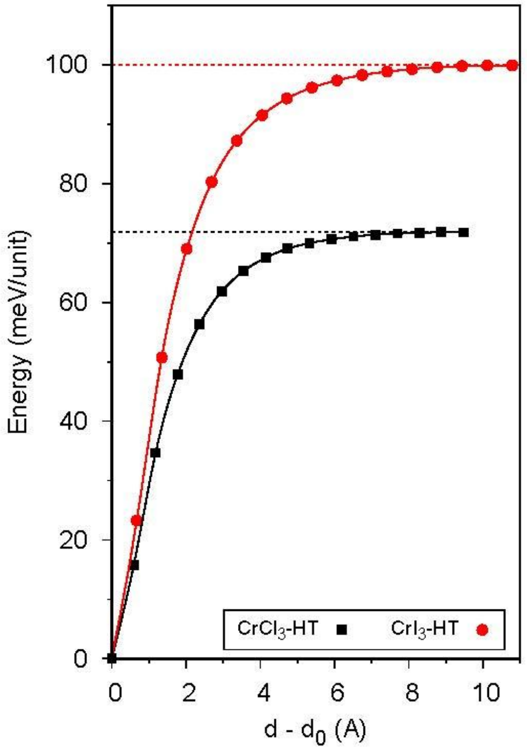

Figure S6. Calculated energy to separate bilayer into two monolayers using DFT. d_0 is the equilibrium interlayer distance and d is the separation between layers of CrX_3.

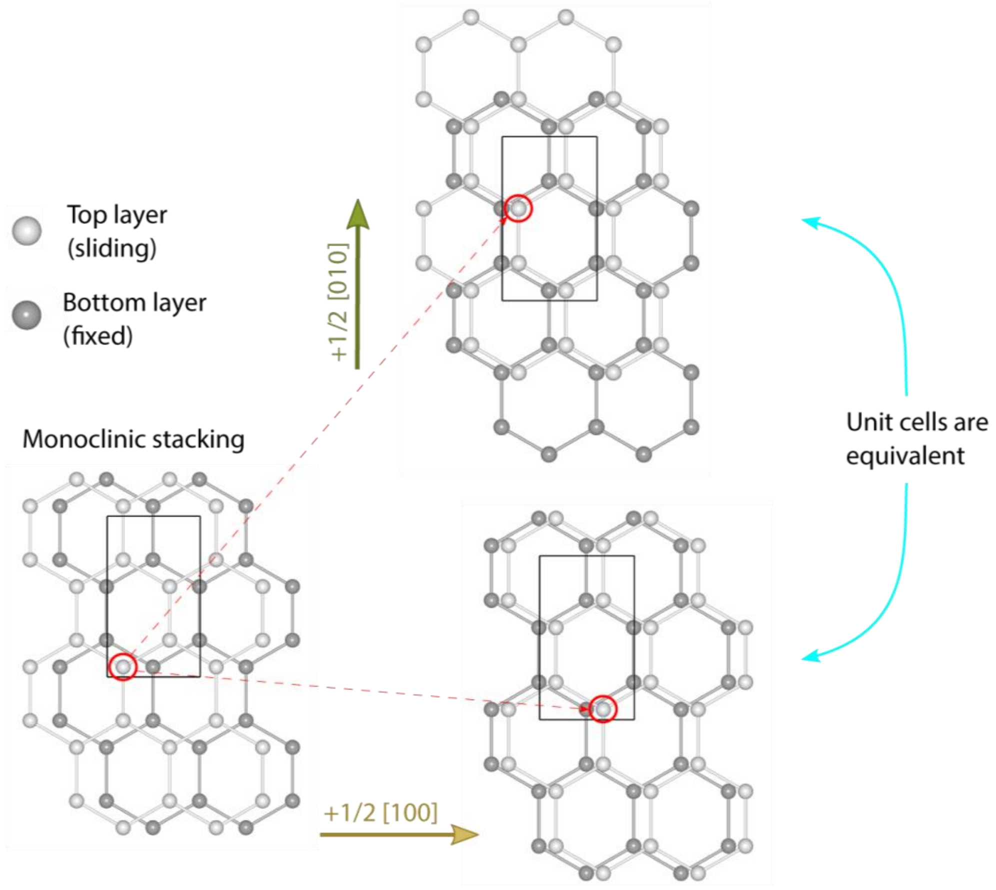

Figure S7. Schematic of the equivalence of sliding process from 1/2 to 2/3 along [100] or [010]. Both situations resulted in similar unit cells.

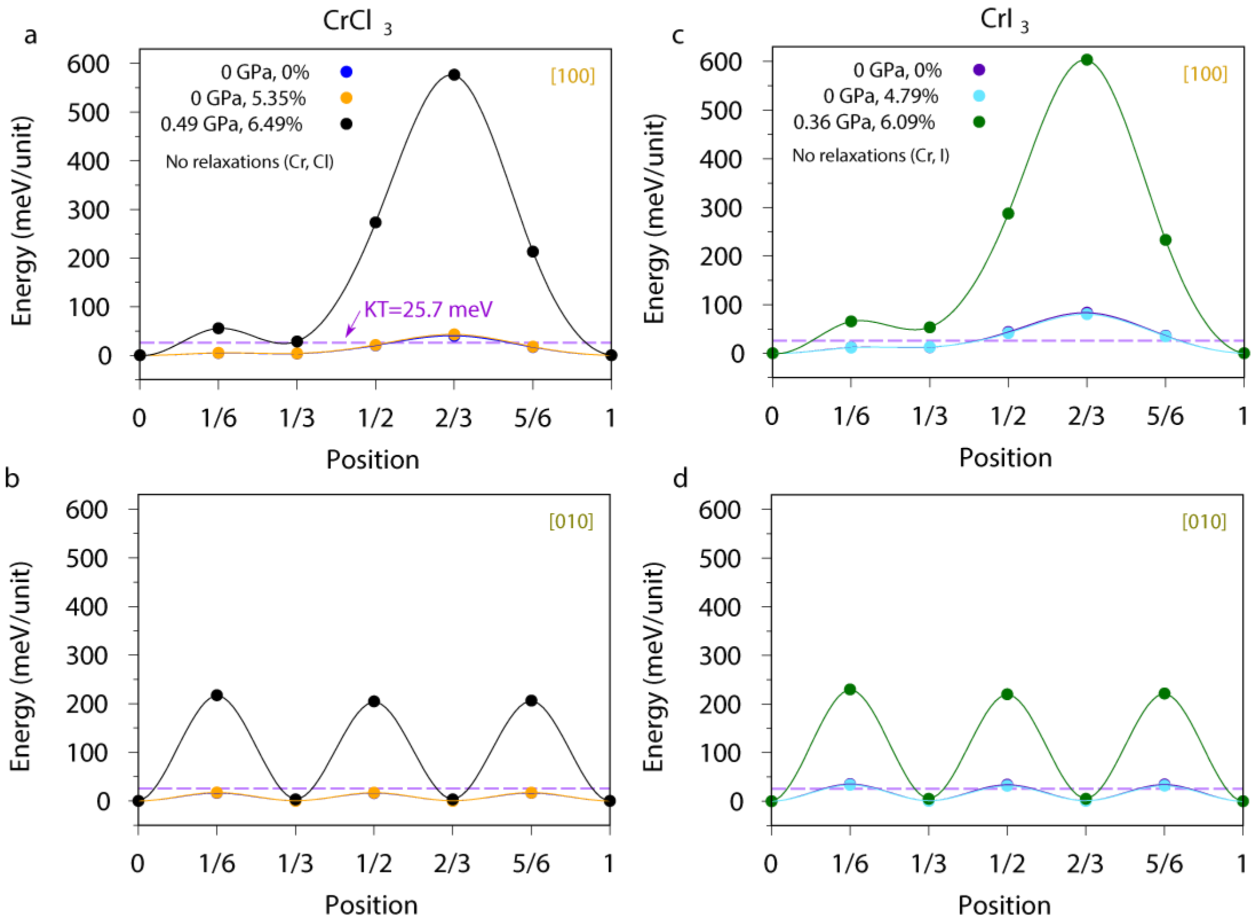

Figure S8. Calculated energy barriers for CrCl3 and CrI3 along [100] and [010]. This is similar as Figure 3 in the main text but with no relaxation performed on the Cr, Cl, I atoms.

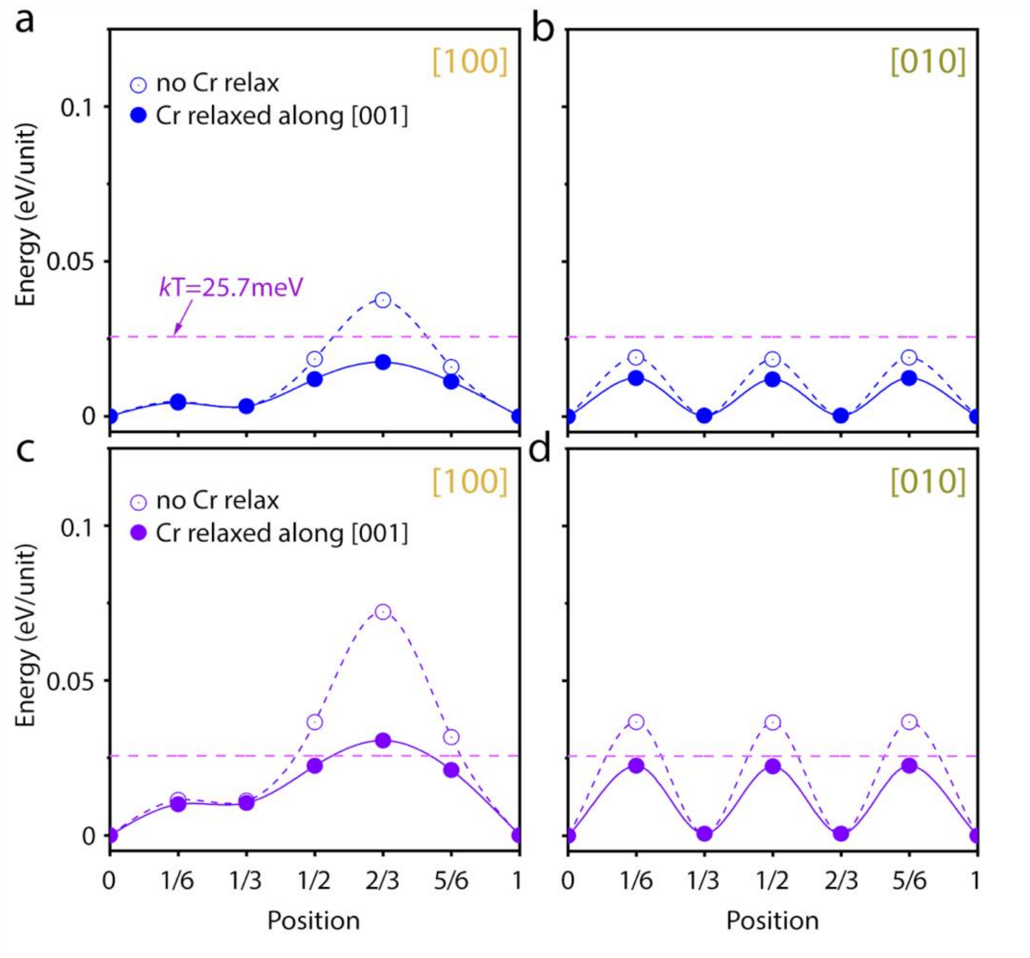

Figure S9. Calculated energy barriers at 0 GPa and 0% strain for $CrCl_3$ (a, b) and CrI_3 (c,d) taking into account relaxations of the Cr atoms at both layers. The halide atoms are previously relaxed as shown in the results Figure 3. The [001] orientation is pointed out of the plane.

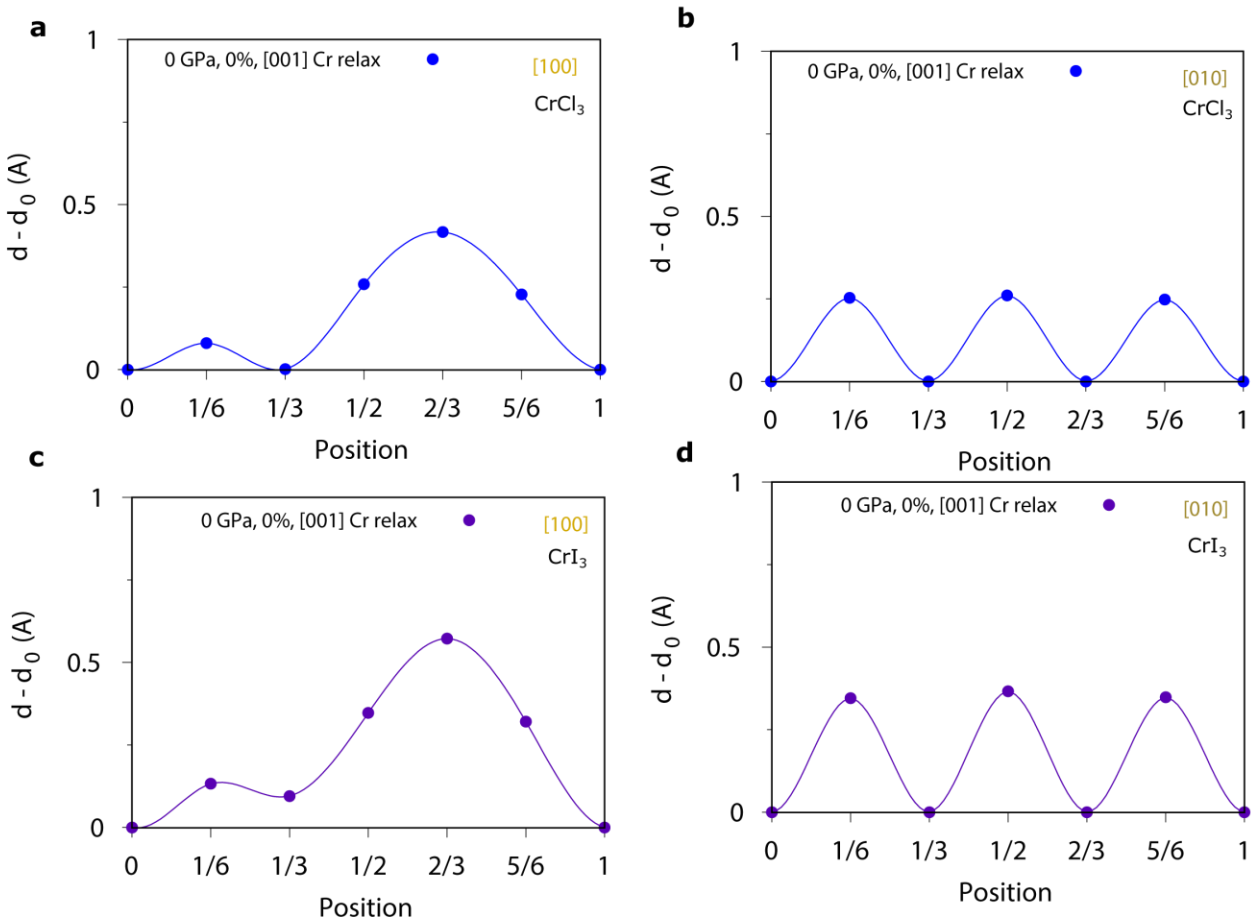

Figure S10: Variation of the interlayer distance at the different positions of the sliding path: along [100] and [010] for $CrCl_3$ (a, b) and CrI_3 (c, d) at 0 GPa and 0% strain. Cr atoms are allowed to relax along the [001] direction. d_0 corresponds to the equilibrium position.

7. Brittle nature of few-layer CrX_3

As proof of the brittle nature of the free-standing CrX_3, Figure S11 provides evidence of reversible load-displacement curves in a free-standing membrane of few-layer CrX_3. In this experiment multiple consecutive indentations were performed in the same membrane avoiding reaching loads, which exceed the rupture point.

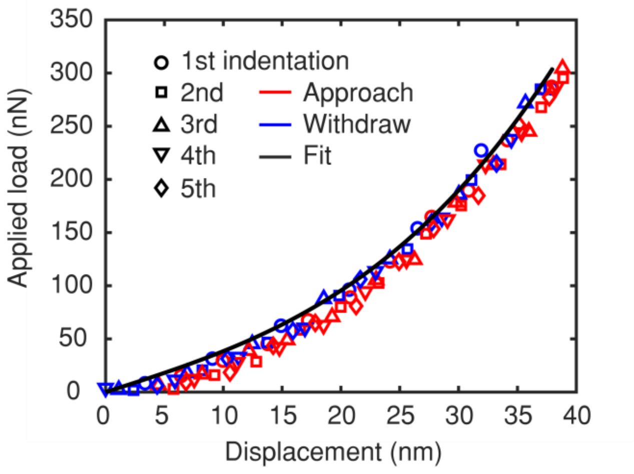

Figure S11. Brittle nature of few-layer $CrCl_3$. Set of five consecutive indentations without fracture on the same membrane ($CrCl_3$ ~ 10 nm). The data points have been down sampled to differentiate between the different indentations. It can be seen that the approach and withdrawal curves follow the same path and are reproducible, indicating that the behavior of the membrane is elastic and not plastic or ductile.

8. Enhanced plasticity in multilayer CrX_3

Material	$\boldsymbol{E_s}$ (meV/f.u.)	$\boldsymbol{E_c}$ (meV/f.u.)	$\boldsymbol{E_{in}}$ (GPa)	$\boldsymbol{\Xi}$ **factor** (GPa^{-1})
$CrCl_3$	19.01500	71.64250	62.1 ± 4.8	0.06104 ± 0.00471
CrI_3	36.68625	99.73875	43.4 ± 4.4	0.06330 ± 0.00642

Table S3. Calculated the deformability factor for CrX_3 materials.

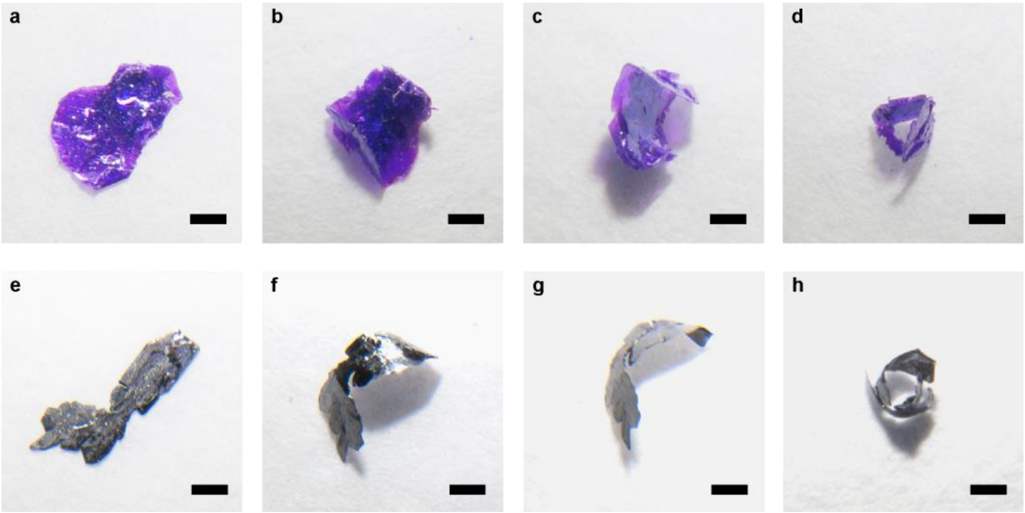

Figure S12. Macroscopic bending test on bulk crystals. a-d, $CrCl_3$, **e-h,** CrI_3. All scale bars are 1 mm in size.

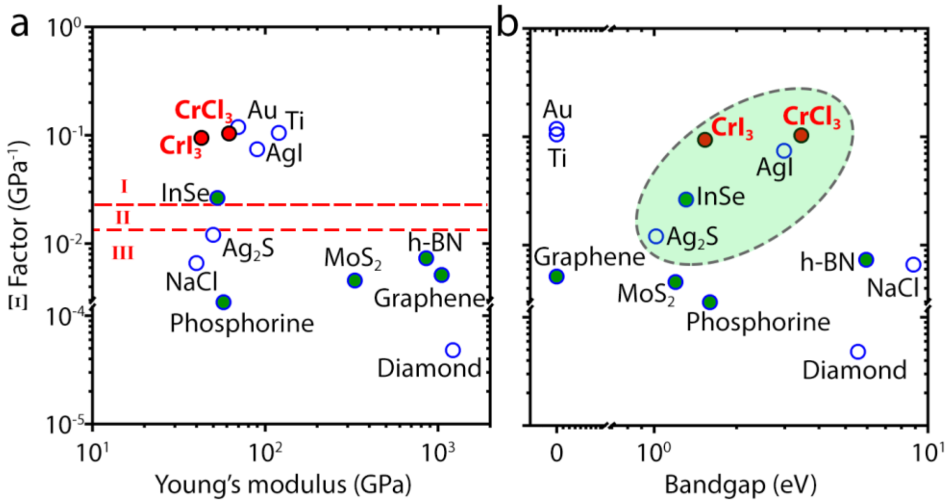

Figure S13. Deformability factor calculated for multilayer CrX_3 with both Cr and halide atoms relaxed during computation. Deformability factor dependence a, on Young Modulus and b, on the bandgap for the same materials. The deformability factor was determined with Cr atoms relaxed along [001] and halides (X = Cl, I) are relaxed in-plane. The legends to the plots are the same with Figure 5 in the main text.
